# Computational Fluid Dynamics Analysis of a Venturi-Integrated Diffuser Design for Membrane Bioreactors

**DOI:** 10.3390/membranes16010010

**Published:** 2025-12-30

**Authors:** Veli Batmaz, Necati Kayaalp

**Affiliations:** 1 Civil Engineering Department, Division of Hydraulics, Engineering Faculty, Şırnak University, 73000 Şırnak, Turkey; 2 Civil Engineering Department, Division of Hydraulics, Engineering Faculty, Dicle University, 21000 Diyarbakır, Turkey; necati.kayaalp@dicle.edu.tr

**Keywords:** air injection, computational fluid dynamics (CFD), membrane bioreactor (MBR), Venturi injector, wastewater treatment

## Abstract

In a standard diffuser system in a membrane bioreactor (MBR), uneven air distribution scouring the membrane surface causes transmembrane pressure to reach its ultimate value earlier, which requires membrane cleaning more frequently. In this study, a Venturi-integrated innovative diffuser design is proposed to improve membrane bioreactor (MBR) technology. The proposed design aims to increase filtration efficiency by creating a homogeneous scouring effect on the membrane surface. To compare the performance of the proposed diffuser configuration (V-MBR) with that of a conventional diffuser (S-MBR), computational fluid dynamics models were established for each of the two configurations. The results showed that the V-MBR model produced about 50% higher average shear stress on the membrane surfaces. Statistical analysis also showed that the V-MBR model generally produced low variance and non-zero shear stress values. Along with shear stress distribution, other parameters such as volume fraction, velocity, turbulent kinetic energy, and turbulent eddy distribution were evaluated to compare the performance of two diffuser system configurations. These parameters also supported the superior performance of the new V-MBR model over the conventional S-MBR. It is concluded that homogeneous shear stress distribution on the membrane surface is an important parameter that increases filtration efficiency by preventing the formation of dead zones.

## 1. Introduction

In recent years, the global demand for drinking water and industrial water has significantly increased due to the rising world population and the urbanization process [1]. This trend has led to an increase in the amount of wastewater discharges. The world’s limited clean water resources are gradually diminishing due to these impacts. Treated wastewater provides a reliable alternative water source, contributing to the alleviation of water scarcity and reducing the demand on freshwater resources [2]. Furthermore, wastewater treatment is of great importance for environmental protection, sustainability of water resources, the safeguarding of public health, and economic development. Wastewater treatment processes eliminate harmful pollutants from water, enabling the reuse of treated water for irrigation, industrial uses, and drinking water. This process ensures compliance with environmental standards and supports sustainable water management by contributing to the efficient use of water resources. All currently available commercial membrane bioreactor (MBR) processes use the membrane ostensibly as a filter. Thanks to this filter, the solids produced by the biological process are rejected, and a purified product is obtained [3]. The use of MBR systems is rapidly increasing due to the obvious advantages of a smaller footprint, better effluent quality, higher organic loading rate, and lower sludge production. However, high operating expenses compared to conventional activated sludge (CAS) technology are a barrier to widespread adoption [4]. The main disadvantage of MBR systems is that frequent cleaning and replacement requirements caused by membrane fouling reduce operational efficiency and increase maintenance expenses. This reduces the attractiveness of MBR technology in full-scale applications [5,6]. Aeration is used as the most efficient membrane cleaning method due to the effective scouring between two different phases (liquid–gas). Although there are many types of aerators, the most common method for aeration tanks in MBR systems is compressed air supply through diffusers below the water surface [7]. However, this method, which is used to increase membrane filtration efficiency, leads to high operating costs [8,9]. Some studies have reported that membrane fouling removal through aeration accounts for approximately 60–80% of total plant energy consumption [10,11]. This high energy consumption reduces the efficiency of MBR technology and therefore hinders its commercialization and widespread use [12]. If we overcome its current disadvantages, MBR technology stands out as a treatment technology with the potential to meet the requirements. When the studies on increasing energy efficiency of MBR systems are examined, it is seen that these studies are generally carried out by arrangement of the aeration regime, modification of the diffuser configuration, optimization of the geometry and layout of the membrane modules, and mechanization of the membrane modules by methods such as rotation and vibration [8]. However, despite the high energy consumption, it is still difficult to achieve homogeneous stress distribution on the membrane surface. This process continues to lead to the formation of dead zones and localized clogging, resulting in low yields.

Some researchers have studied bubble formation with Venturi devices due to their simple structure, potential to enhance interphase mass transfer, and especially its energy consumption efficiency. In [13], different MBR configurations were studied, where the Venturi injector is connected both in parallel and in series, creating a suction pressure in the throat section through a circulation line. In another study, laboratory-scale experimental studies were conducted by integrating a Venturi injector into a submerged MBR, where a standard diffuser was previously used, and it was reported that the Venturi injector simultaneously increased both gas transfer efficiency and membrane surface cleaning efficiency [14,15]. According to [16], while a standard bubble diffuser system achieves an O_3_ mass transfer efficiency of about 10–15%, a system using a Venturi injector achieves a mass transfer efficiency of 90%. In a study by [17], a new micro-Venturi apparatus for wastewater treatment processes was designed and integrated into the reactor by optimizing its structural parameters. The researchers reported a 61% reduction in chemical oxygen demand (COD) after three hours of treatment to evaluate the effect of the Venturi injector on aeration efficiency. In [18], a new Venturi injector was designed for the biological treatment of wastewater containing ammonia nitrogen, and the performance of this design was evaluated both experimentally and numerically. With the new Venturi design they developed, the researchers produced small and homogeneous air bubbles with a diameter of 0.71 mm and above, achieving 2 times higher ammonia removal efficiency compared to the standard aeration system and 1.1 times higher ammonia removal efficiency compared to the standard Venturi system. These studies have focused on the aeration capacity and oxygen transfer efficiency of the Venturi injector. However, membrane fouling processes such as plugging and flux reduction caused by organic and inorganic particles that can accumulate on the membrane surface have not been discussed. Therefore, comprehensive studies need to be conducted to determine the membrane fouling reduction capacity for Venturi-MBR integration and to identify the optimum configurations.

Computational Fluid Dynamics (CFD), a powerful analysis tool, has been used by many researchers to study physical scour methods and predict flow dynamics in MBR systems. CFD can predict the effects of air bubble type, membrane module configuration, and other design parameters on fluid dynamics at a fundamental level [8]. CFD tools are widely used either to successfully design and operate treatment systems with better hydraulic flow patterns and high pollutant removal efficiencies or to improve existing treatment systems to achieve higher performance with lower energy consumption [1]. It also offers the opportunity to simulate, an important advantage when real experiments can be time-consuming and costly. So far, CFD studies have contributed significantly to the regulation of aeration regimes in MBR systems and to a deeper understanding of fouling mechanisms.

**Figure 1:**
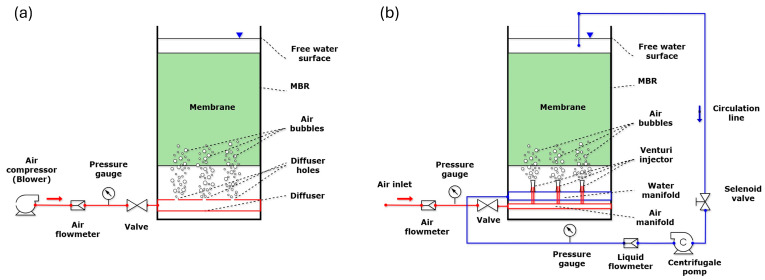
Comparative schematic representation of the computational models used in the analysis showing the structural differences between (**a**) S-MBR and (**b**) V-MBR.

In this study, the air used in membrane cleaning processes in MBR systems is provided by an innovative diffuser design with integrated micro-Venturi instead of a standard diffuser. Standard diffusers, despite consuming large volumes of gas, are insufficient to create a homogeneous scouring effect on the membrane surface. Therefore, this design aims to improve the performance of the diffusers and create a homogeneous scouring effect on the membrane surface. In the new design, micro-Venturis are vertically integrated into diffusers in the reactor bottom, directing the high-energy air–water mixture to the membrane modules. The energy to create suction pressure in the throat section of the Venturi injectors is provided by a circulation line. The focus of the study is the numerical comparison of models of a membrane bioreactor (S-MBR) with a standard diffuser and a micro-Venturi injector-integrated membrane bioreactor (V-MBR) (Figure 1). For this purpose, firstly, the S-MBR model was reconstructed based on the S-MBR geometry and operating conditions presented in [19]. Secondly, the V-MBR model was developed with design modifications to the diffuser structure. Thirdly, both models were analyzed using the CFD analysis tool ANSYS Fluent 2021 R1 software, and the results obtained were compared. This comparison aims to evaluate the effect of the design change in the diffuser structure on the flow dynamics in the MBR by the numerical modeling method.

## 2. Materials and Methods

### 2.1. Model Geometry

**Figure 2:**
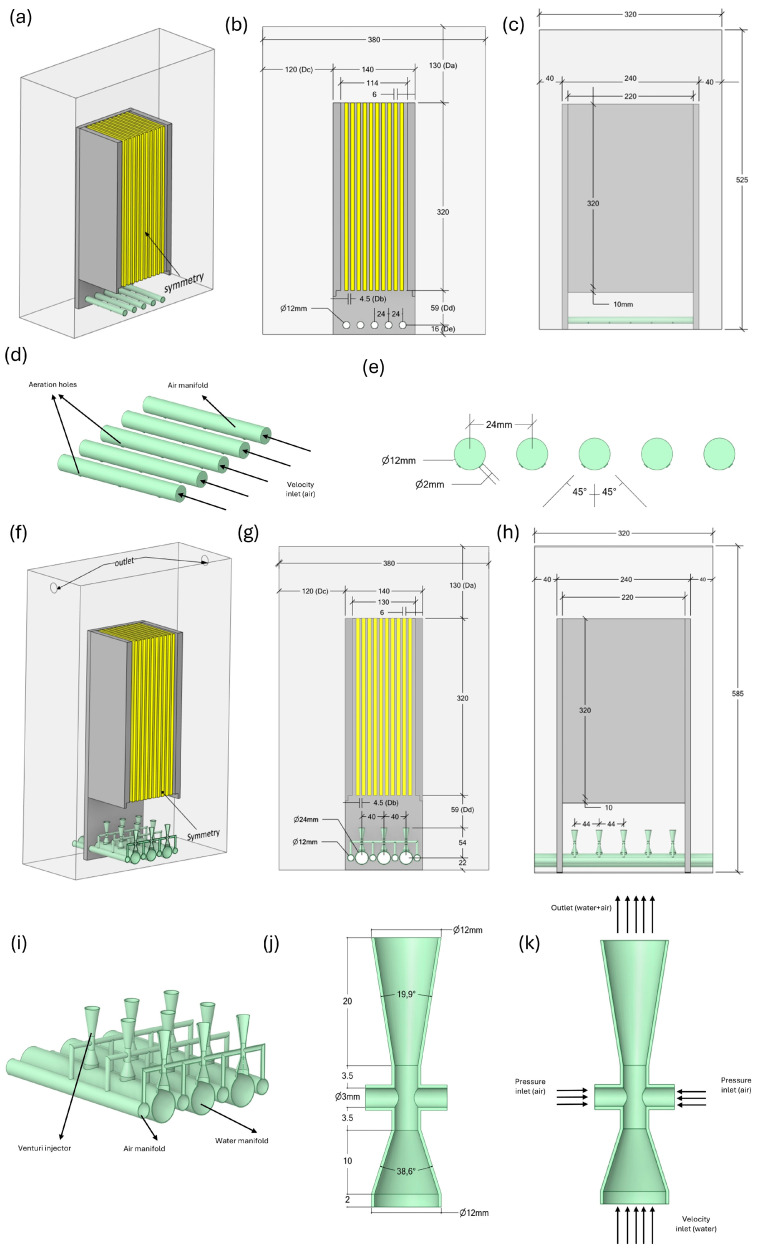
Geometry aspects of the models employed in the analyses: (**a**–**c**) S-MBR; (**d**,**e**) air manifold for S-MBR; (**f**–**h**) V-MBR; and (**i**–**k**) Venturi-integrated diffuser for V-MBR.

The membrane module geometry, which is optimized for an S-MBR in [19], was used for the numerical analyses in this study. The following is a quick summary of the feature specifications of the S-MBR model that was examined in [19]. In the reactor ten flat-sheet (FS) membrane modules (L × W × H = 320 mm × 220 mm × 220 mm × 6 mm) were positioned parallel to one another and spaced 4.5 mm apart. The ideal reactor dimensions were found to be L × W × H = 380 mm × 320 mm × 525 mm as a result of optimization. The membrane modules were positioned beneath five air manifolds (L × d = 240 mm × 12 mm). Eleven aeration holes, each measuring 2 mm in diameter, were positioned on the underside of each air manifold, in both directions at a 45° angle to the vertical. To lessen sludge intrusion into the diffuser, the aeration holes were positioned at the bottom (Figure 2).

Except for the diffuser structure, the geometrical properties of the newly proposed V-MBR configuration are identical to those of the S-MBR model detailed in reference [19]. This is desired to make the comparison of our study’s numerical analysis findings to the aforementioned study more meaningful. Instead of the typical diffuser utilized in [19], a novel diffuser design with an integrated micro-Venturi injector was employed. The reactor base features three water manifolds (Lw × dw = 320 mm × 24 mm) spaced 40 mm apart. Each manifold had 5 micro-Venturi injectors vertically connected at 44 mm intervals, and the membrane modules received a total of 15 micro-Venturi outlets. Design parameters such as the diameters of the Venturi injector’s inlet and outlet ports, as well as convergence and divergence angles, were determined using the literature’s ideal Venturi geometry and the MBR dimensions we employed. The Venturi injector’s inlet and outlet ports were 12 mm in diameter, with a convergent cone angle of 38.6° and a divergent cone angle of 19.9°. To boost the air suction capacity of the Venturi injectors, a 3 mm diameter suction port was installed on both sides of the throat. This design aims to optimize the system’s air distribution and achieve a more uniform flow profile on the membrane surface. The suction ports connect to the atmosphere by four air manifolds (La × da = 320 mm × 12 mm) located between the water manifolds. After integrating the Venturi injectors, the new dimensions changed to L × W × H = 380 mm × 320 mm × 585 mm (Figure 2).

In this study, the hydrodynamic behaviors of the flow in S-MBR and V-MBR models are thoroughly explored using ANSYS Fluent 2021 R1 software. To optimize computational efficiency, both models’ computational domains were decreased utilizing symmetry axes. Figure 2 illustrates the geometrical features and design specifics of the S-MBR and V-MBR variants.

### 2.2. Operating Principle of the Venturi Injector

Venturi tubes are simple but effective hydraulic devices based on Bernoulli’s principle and composed of three basic sections: the converging cone, the cylindrical narrow section (throat section), and the diverging cone. These devices can also be used to measure the flow rate of liquid in a pipeline. The narrowing of the cross-section through which the fluid flows increase the flow velocity and reduces the pressure, and this pressure difference is used to calculate the flow velocity. Venturi tubes are widely preferred in engineering applications due to their low energy loss and high measurement accuracy.

**Figure 3:**
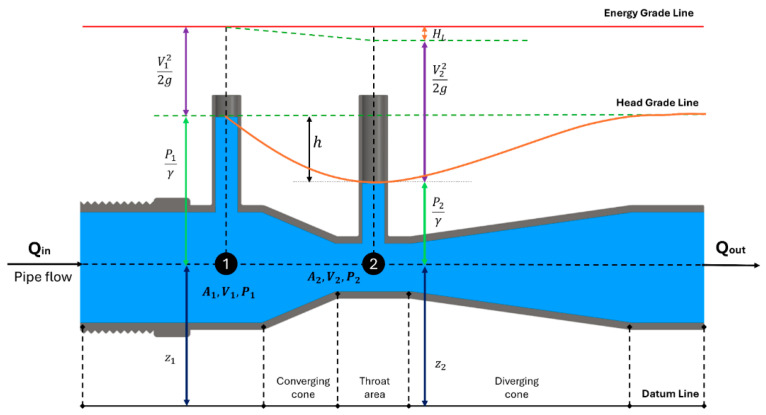
Theoretical variables of a Venturi device.

The energy equation between Section 1 and Section 2 in Figure 3 can be expressed as follows:

(1) $$\frac{P_{1}}{\gamma }+\frac{V_{1}^{2}}{2g}+z_{1}=\frac{P_{2}}{\gamma }+\frac{V_{2}^{2}}{2g}+z_{2}+H_{L},$$

where $P_{1}$ and $P_{2}$ represent the pressures (N/m^2^) at Section 1 and Section 2, $z_{1}$ and $z_{2}$ are the heights of these points relative to the datum line (m), $H_{L}$ is the head loss (m), and γ is the specific gravity of water (N/m^3^). The equation can be written in the following form to state the pressure difference between the two sections:

(2) $$\left(P_{1}-P_{2}\right)=\gamma \left(\frac{V_{2}^{2}}{2g}-\frac{V_{1}^{2}}{2g}+H_{L}\right),$$

According to the steady-state form of the continuity equation, the amount of mass per unit time entering and leaving any two sections of the pipe must remain constant. For fluids that are considered incompressible, such as water, the density is taken as constant, and this is expressed as follows:

(3) $$Q=V_{1}A_{1}=V_{2}A_{2},$$

where $V_{1}$, $V_{2}$, $A_{1}$, and $A_{2}$ represent the average cross-sectional velocities and cross-sectional areas at Section 1 and Section 2, respectively. In the throat section, there is an increase in velocity (${V_{2}>V}_{1}$) due to the narrower cross-sectional area ($A_{2\;}$<${\;A}_{1}$). In parallel with the increase in velocity, there is a decrease in pressure ($P_{2}\;$<${\;P}_{1}$). In the divergent region, velocity is converted back to pressure with some loss ($H_{L}$). This indicates that the pressure at point 2 has become negative relative to the reference pressure at point 1. Negative pressure indicates that the static pressure has fallen below atmospheric pressure. When sufficient flow velocity occurs in the Venturi device, the pressure in the throat section leads to negative gauge pressure (vacuum). This negative gauge pressure draws the liquid or gas connected to the throat section (suction port) and mixes it with the working fluid [4]. In this study, this simple working principle of Venturi tubes is utilized for air injection into the MBR tank. The potential of air injected into the MBR with micro-Venturis integrated into the standard diffuser to clean the membrane surface was investigated with a new design.

### 2.3. Meshing

In computational fluid dynamics (CFD) modeling, the proper definition of the grid structure of the flow domain is perhaps the most critical step in the solution process. Therefore, the optimal grid structure should be determined by balancing the mesh quality parameters, computational cost, and convergence criterion of the solution. In the models used in this study, symmetric axes were used to reduce the number of computational cells, and half of the flow domain was simulated. Furthermore, a mesh independence test was performed to determine the optimal mesh size [20,21] because a quality mesh directly affects simulation time and convergence stability [22]. Among the criteria for evaluating mesh quality, the aspect ratio, which indicates the elongation character of the element, starts at 1 for an ideal shape and goes up to ∞ as the shape deteriorates. Skewness, which refers to the distortion of the shape of the element, starts at 0 for an ideal shape and reaches 1 as the skewness increases. A skewness value exceeding 0.95 leads to invalid modeling results. Mesh quality parameters should be within acceptable ranges; otherwise, convergence difficulties and invalid results in terms of accuracy will occur.

Fluent Meshing improves computational efficiency by creating higher resolution and quality mesh structures in a short time with polyhedral and polyhex-core mesh structures, especially for flow problems with complex geometries. These methods increase numerical accuracy and computational speed by more accurately capturing sharp transitions in the flow domain while optimizing the number of cells. Due to these advantages, a polyhedral mesh was applied to the flow domain with Fluent Meshing. The regions close to the diffuser and between the membranes are critical areas where the flow hydrodynamics change. Therefore, these regions are discretized with smaller mesh elements than the other regions.

In turbulent flows, the velocity and stress distributions near the wall exhibit significantly larger gradients than the remainder of the flow field. Thus, appropriate modeling of the wall region is crucial in the numerical solution of turbulent flows. The dimensionless distance $y^{+}$ defines the mesh resolution near the wall as follows:

(4) $$y^{+}=\frac{\rho u_{T}y}{\mu }$$

Here, *y* represents the distance from the wall surface to the center of the adjacent cell, $u_{T}$ represents the wall slip velocity, ρ represents the fluid density, and μ represents the fluid dynamic viscosity. $y^{+}$ is the fundamental parameter that determines the region in which the wall functions are valid. In this study, the standard k–ε turbulence model was preferred due to its numerical robustness, lack of requirement for excessively fine meshes near the wall, and low computational cost. The k–ε turbulence model uses wall functions based on the logarithmic velocity profile rather than directly solving the flow structure near the wall. The $y^{+}$ range recommended in the literature for this model is 30 < $y^{+}$ < 300 [23]. Numerical calculations performed under the selected mesh structure and flow conditions revealed that the $y^{+}$ value is approximately 35. In this context, the mesh size defined for the wall region and the calculated $y^{+}$ value are compatible with the selected turbulence model.

Although a mesh structure with a larger number of elements produces more accurate simulation results, it causes a higher computational cost and time. To reduce computational costs and time, the number of mesh elements needs to be optimized. The mesh independence test described in Table 1 was used to guarantee that the results were mesh independent.

**Figure 4:**
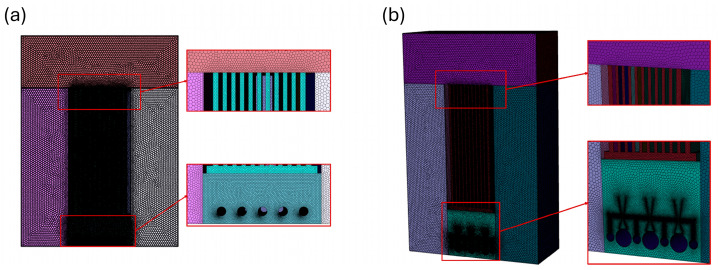
Representation of the mesh structure of the polyhedral used in the analyses for the (**a**) S-MBR and (**b**) V-MBR models.

As a result of the mesh independence test, increasing the number of mesh elements and improving the quality parameters led to an increase in the average shear stress value on the membrane surface. However, doubling the number of mesh elements means that the computational cost will also increase by about a factor of two. Despite doubling the number of network elements when transitioning from Model 5 to Model 7, only a limited increase of 0.54% was observed in the average shear stress value, while a decreasing difference of 2.89% was detected in the average velocity values obtained along the symmetry axis. Considering the computational cost, negligible differences in the results, and mesh quality parameters, model 5 was determined to be the optimum mesh structure to be used in the analysis (Table 1). With the mesh properties determined, a polyhedral mesh structure was created for the S-MBR and V-MBR models, and comparative analyses were performed (Figure 4).

### 2.4. Model Setup

To reduce the computational cost, researchers often use simplifications. These include modeling three-phase flows (gas–liquid–solid) as two-phase flows (gas–liquid) and assuming bubbles stay the same size [24,25]. In this study, the activated sludge system is simplified as a two-phase flow, consisting of water and air, and the effects of particles on the flow are ignored. It is also assumed that the bubbles created by aeration are spherical. However, we do not have experimental data on the size of the air bubbles in the V-MBR model. To facilitate comparisons and mitigate the influence of bubble size variations, a bubble diameter of 5 mm was employed in both models. The Euler model was selected for the numerical simulation, given its capacity to efficiently simulate gas–liquid flow and its appropriateness for the research objectives. Furthermore, the standard k–ε turbulence model was implemented to simulate turbulent flow; this model is extensively utilized in equation-based simulations, owing to its computational efficiency, robustness, and accuracy [26].

The simulation methods, operating conditions, and boundary conditions used in [19] for the S-MBR model were to be the same as the S-MBR model reconstructed for comparison. However, in the S-MBR model, only air is injected into the reactor by diffusers, while in the V-MBR model, a mixture of air and water is injected into the reactor by Venturi injectors. Due to this difference, the boundary conditions for the V-MBR model are defined as follows. The water flow introduced into the reactor to establish negative pressure within the Venturi injector’s throat is classified as a “velocity inlet,” given that its flow rate is regulated by pumps. This methodology facilitates a realistic numerical modeling of the Venturi effect by accurately simulating the necessary velocity augmentation and the corresponding pressure reduction within the throat. Conversely, the air that is drawn into the system through atmospheric openings, driven by the negative pressure generated in the throat, is designated a “pressure inlet,” as its entry into the system is dictated by pressure differentials rather than a predetermined velocity. The specified boundary condition simulates the natural intake of atmospheric air into the reactor, a process driven by the Venturi effect, thereby enabling the air flow rate to evolve autonomously, contingent upon the prevailing pressure distribution within the flow field. This particular set of boundary conditions aligns directly with the operational realities of full-scale MBR systems, where liquid flow is regulated by pumps and aeration is governed by pressure differentials. In addition, the top of the reactor was recognized as a “degassing” to allow air to exit it. The circulation line’s outlet ports were designated as “pressure outlet” for the discharge of the incoming water. The flow velocity as water permeates through the membrane pores is negligibly small compared to the flow velocity of the water-air mixture used to scour the membrane surface. As a result, the effect of the membrane filtration process on the dynamics of the scouring process is ignored, and the membrane surfaces are represented as impermeable fixed walls for both models [27,28]. Table 2 shows the updated V-MBR model parameters that resulted from the aforementioned differences.

## 3. Results and Discussion

### 3.1. Comparison of Hydrodynamic Behaviors in Models

Using the same geometrical parameters, boundary conditions, and operating parameters, the same results as in [19] were obtained for different air velocities in the current study. This indicates that the model presented in [19] was accurately reestablished. In the model presented in [19], based on the total fluid volume supplied to the system for air injection velocities ranging between 1 and 7 m/s, equivalent water flow velocities were calculated to provide the same flow volume in the V-MBR model. The aim here is to investigate the differences that the same fluid flow rate may cause in the S-MBR and V-MBR models in terms of shear stresses.

Higher air and water phase velocities are known to increase shear stress and turbulence at the membrane surface, reducing the risk of membrane fouling [29]. The velocity gradient is directly related to the amount of energy required to reach a certain level of turbulence in the system [28]. Although high inlet velocities increase the energy demand, they do not guarantee a homogeneous shear stress distribution on the membrane surface; on the contrary, a non-uniform distribution is often observed on the membrane surface.

**Figure 5:**
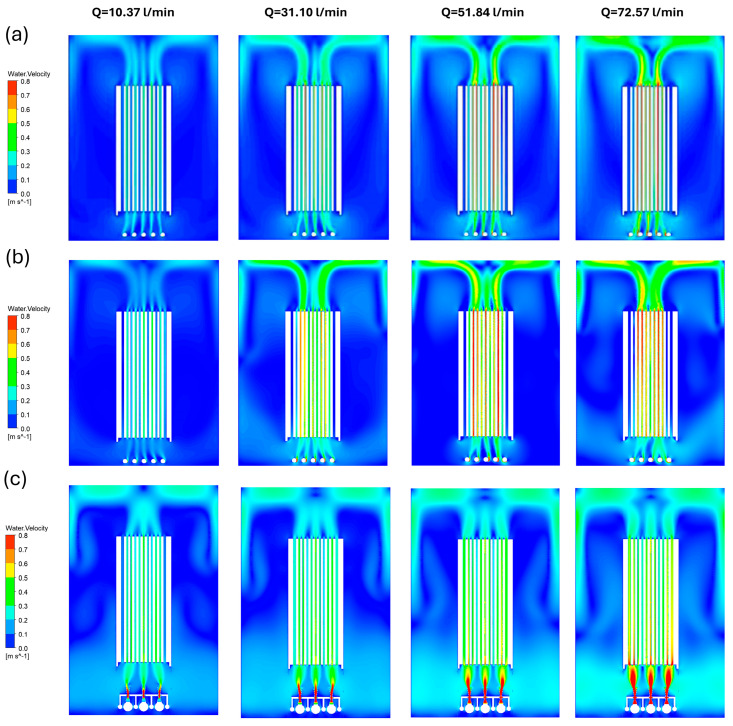
Comparison of water velocity contours on the symmetry plane for various volumetric inflow rates (L/min): (**a**) S-MBR results reported in [19]; (**b**) S-MBR results obtained in the present study under identical operating conditions; (**c**) V-MBR results obtained in the present study.

This is because the flow velocities in certain regions are much higher than in other regions on the membrane surface. Images obtained from membrane fouling studies in the literature also confirm this. Therefore, the homogeneity of the velocity distribution is of great importance as well as obtaining high velocity values on the membrane surface with minimum energy. Homogeneous velocity distribution will prevent the formation of dead zones by creating homogeneous shear stress on the membrane surface and increase the efficiency of the system. In this study, the velocity contours of the S-MBR and V-MBR models are compared in the same symmetry plane because of the analysis under different volumetric inlet flow rates. The velocity contours obtained determine the acceleration and deceleration regions of the flow and enable the detection of turbulent and dead flow regions. The velocity contours of the S-MBR model were found to be consistent with the velocity contours of the model presented in [19]. In the V-MBR model, the velocity distribution between the membrane channels was observed to be more homogeneous compared to the S-MBR model. This difference in velocity distribution observed in the V-MBR model can improve the overall efficiency of the system by producing homogeneous shear stress with a more balanced flow dynamic (Figure 5).

**Figure 6:**
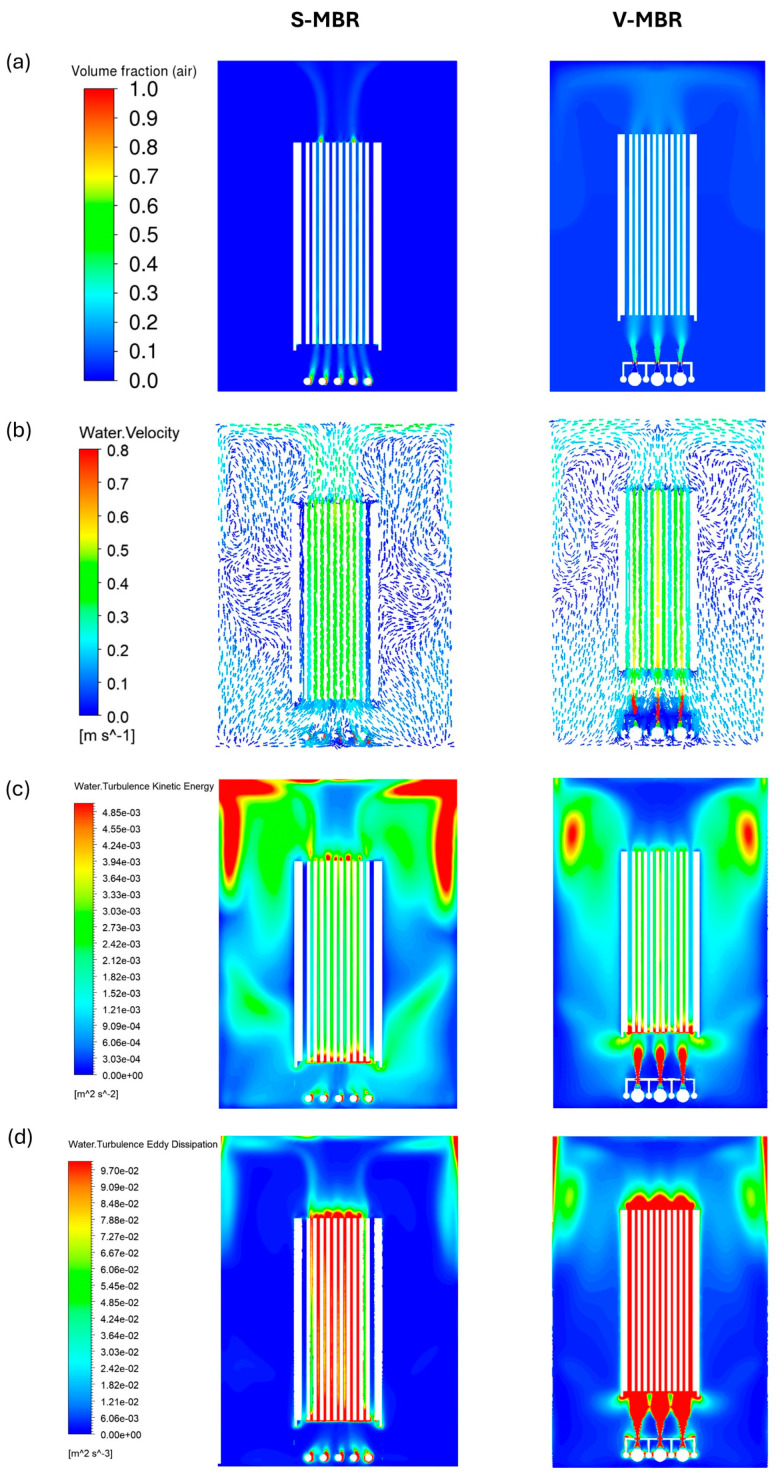
Numerical simulation results comparing the S-MBR and V-MBR systems on the symmetry plane: (**a**) air volume fraction distribution showing gas holdup and bubble distribution behavior inside the reactor; (**b**) water velocity vectors indicating flow direction and magnitude; (**c**) water turbulent kinetic energy distribution highlighting regions of high turbulence; (**d**) water turbulent eddy dissipation explaining the spatial variation in turbulence diffusion.

The numerical results obtained with the same volumetric fluid inflow rate (31.10 L/min) are presented in Figure 6 for the S-MBR and V-MBR models. Figure 6a shows that in the S-MBR model the air bubbles move in a narrower area between the membrane channels, whereas in the V-MBR model the air bubbles are distributed over a wider area with Venturi injectors directed under the membrane. This difference in the V-MBR model increases the air–water interaction and helps create a homogeneous turbulence distribution over a large area. In Figure 6b, in the S-MBR model, the water velocity appears relatively homogeneous across the membrane, and vortex formation is more limited in the lower region. In contrast, in the V-MBR model, the water velocity is higher in the lower region of the membrane, and this leads to significant vortex formation in the inlet region. The velocity vectors are more pronounced, especially in the channels in line with the Venturi outlets, and these characteristics may favor the removal of particles accumulated on the membrane surface and increase the filtration efficiency.

In Figure 6c, the turbulence kinetic energy shows a similar distribution in the lower region of the membrane in both models. However, a lower level of turbulence kinetic energy is observed in the edge membrane channels in the S-MBR model. Figure 6d shows that for the S-MBR model, the turbulence eddy distribution on the membrane surface is low, especially on the edge membranes and in the regions below the membrane. In contrast, for the V-MBR model, a higher eddy distribution occurs in the lower regions of the membrane and on all membrane surfaces. This may promote the removal of particles from the membrane surface by increasing turbulent mixing.

### 3.2. Comparison of Shear Stress Distributions on the Membrane Surface

The purpose of this study is to compare the shear stress on the membrane surface resulting from integrating the Venturi injector, which has been shown in the literature to have a high oxygen transfer rate, into the MBR system to the shear stress obtained in a conventional submerged MBR using a typical diffuser. Shear stress is a shear force caused by fluid (water, air, or water + air) movement on the membrane surface. Shear stress helps to remove particles and contaminants that have accumulated on the membrane surface. As a result, the shear stress values on the membrane surface of the S-MBR and V-MBR models derived from the analysis utilizing different volumetric flow rates are thoroughly compared in this section.

**Figure 7:**
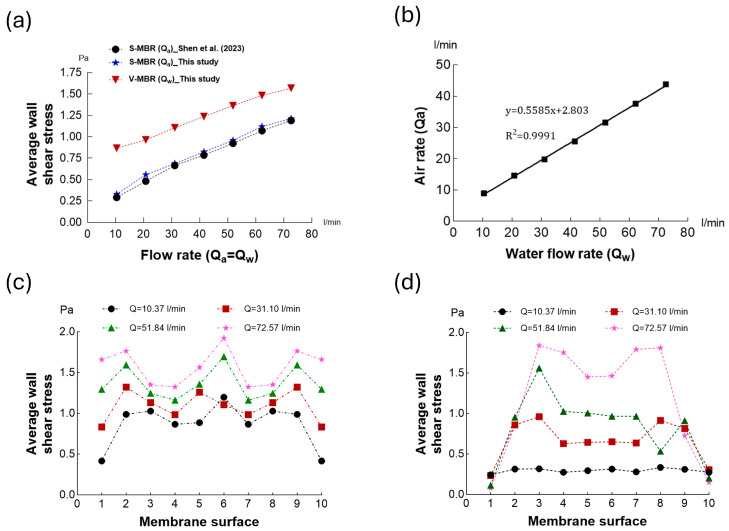
(**a**) Comparison of the V-MBR and the re-designed S-MBR models in this study with the S-MBR reported in [19] under the same conditions. (**b**) Correlation between the volumetric air flow rate (L/min) injected through the Venturi suction port and the volumetric water flow rate (L/min) entering the V-MBR model. (**c**) Comparison of the average shear stress on the membrane surface for different volumetric water flow rates in the V-MBR model. (**d**) Comparison of the average shear stress on the membrane surface for different volumetric water flow rates in the S-MBR model.

In Figure 7a, the average shear stresses on the membrane surface for the S-MBR and V-MBR models for different volumetric flow rates are compared with the average shear stresses previously obtained for the S-MBR [19]. When reproducing the findings of Ref. [19], the results were on average 4% higher than the values presented by the researchers. This difference is thought to be mainly due to the different mesh structures used in the models. Especially for diffuser holes that provide air inlet, the “curvature” and “proximity” settings should be turned on, and the minimum element size should be determined to clearly capture these surfaces. Otherwise, small surfaces such as diffuser hole diameters are not captured clearly. This leads to the inclusion of a volume flow rate different from the analytically calculated volume flow rate. In this study, the effect of mesh refinements on the average shear stress on the membrane surface was investigated using the mesh independence test (Table 1). Utilizing result of the mesh independence test, the optimum mesh structure was determined, and all analyses were performed accordingly.

The average shear stress obtained with the V-MBR model showed a significant increase of about 50% compared to the S-MBR model. For the V-MBR model, the relationship between different volumetric water flow rates and the volumetric air flow rate injected through the Venturi suction port is presented in Figure 7b. As a result of the simple linear regression, the coefficient of determination (R^2^) was found to be very close to 1 as predicted. This indicates that the amount of air injected is proportional to the water inlet velocity and that high efficiency can be achieved at high flow velocities in full-scale systems. The comparison of the average shear stresses on the membrane surface with different volumetric flow rates in the V-MBR and S-MBR models is presented in Figure 7c and Figure 7d, respectively. It is seen that the average stress value increases as the volumetric inlet flow rate increases in the S-MBR model. However, despite the increase in the inlet flow rate, close and low shear stress occurred on the membrane surfaces at the edge regions (1, 2, 9, 10). This phenomenon may lead to the inability to obtain the desired efficiency in proportion to the increase in the energy consumed in the S-MBR model. In the V-MBR model, it is seen that the average stress value increases as the volumetric inlet flow rate increases, and a more homogeneous stress distribution occurs compared to the S-MBR model.

**Figure 8:**
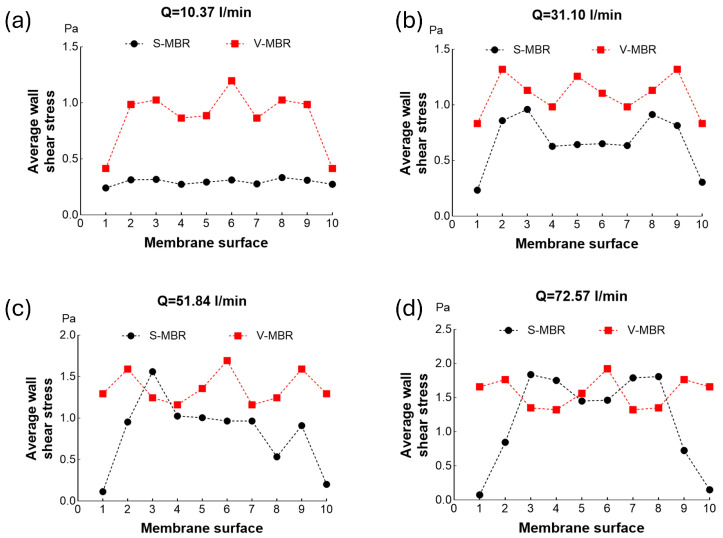
Comparison of the average shear stress on the membrane surfaces for S-MBR and V-MBR at different volumetric inflow rates: (**a**) 10.37 L/min; (**b**) 31.10 L/min; (**c**) 51.84 L/min; (**d**) 72.57 L/min.

The shear stresses at the membrane surface of the S-MBR and V-MBR models are compared separately for different volumetric inlet flow rates in Figure 8. In the S-MBR model, a homogeneous shear stress distribution is observed at a low volumetric air inlet flow rate (10.37 L/min). However, as the volumetric inlet flow rate increases, the shear stress homogeneity is disturbed and concentrated in the central regions. On the contrary, in the V-MBR model, at low volumetric water inlet flow rate (10.37 L/min), low shear stresses were detected in membranes 1 and 10, which disrupted the homogeneity. However, with increasing volumetric inlet flow rate, homogeneity was largely restored.

The S-MBR model’s inhomogeneous distribution of shear stress poses a serious difficulty. Ref. [3] noted that aeration uniformity is crucial for controlling membrane fouling in industrial-scale membrane modules, while [30] was successful in boosting the peak shear stress value in the bulk solution to 40 Pa but noted that it is difficult to generate a comparably high and uniform shear stress distribution in the film region between the bubble and the membrane surface. According to [31], slug flow is effective for fouling control of submerged flat sheet MBR modules; however, it is not favored due to the unequal bubble distribution, which results in an inhomogeneous shear stress profile across the membrane. The results of this study reveal that the V-MBR model surpasses the S-MBR model in terms of generating homogeneous shear stress.

**Figure 9:**
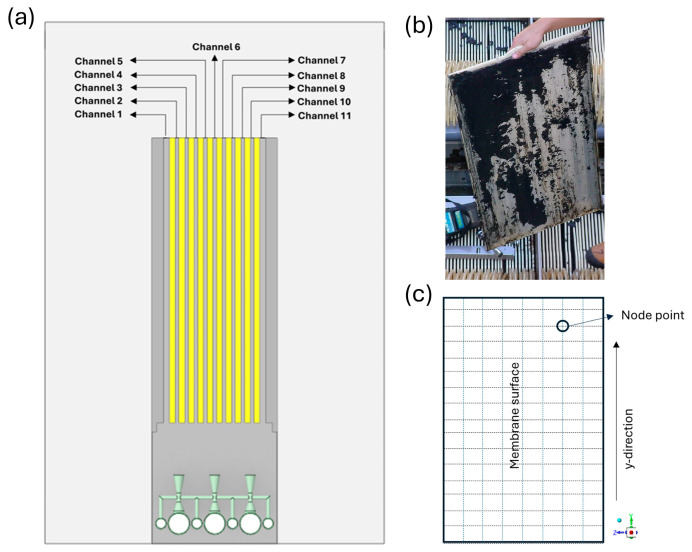
(**a**) Schematic representation of the membrane channels for the V-MBR and S-MBR models. (**b**) Clogging of MBR membrane channels in a flat-sheet module [32]. (**c**) Schematic depiction of the membrane surface used to calculate shear stress at a node point.

**Figure 10:**
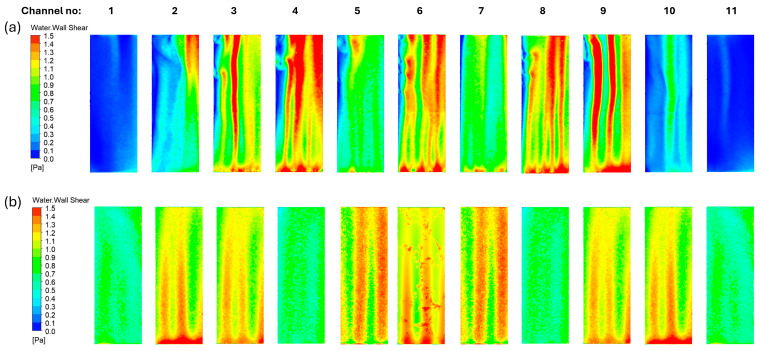
Comparison of shear stress distributions in membrane channels: (**a**) shear stress contours obtained in the S-MBR model; (**b**) shear stress contours obtained in the V-MBR model.

The analysis focuses on the homogeneous shear stress distribution on the membrane surface, which is an important parameter that has a direct impact on system performance. In fact, homogeneous shear stress distribution implies that the average shear stress throughout each membrane, as well as the shear stress at all points on the membrane’s surface, are closely related. As a result, calculating the average shear stress at each membrane surface is insufficient to assess the outcomes. To do this, the average shear stress contours in the membrane channels depicted in Figure 9a were constructed and compared in the range of 0–1.5 Pa for the S-MBR and V-MBR models, respectively, with an ideal volumetric inlet flow rate of 31.10 L/min (Figure 10). In the S-MBR model, minimal shear stresses are detected in membrane channels at the edges. The membrane channels in the center sections produce strong shear stresses in specific directions, whereas low shear stresses are also recorded on the same surface. In other words, while close to other membrane surfaces, low and high shear stresses coexist on the same surface. These conditions can cause complete clogging of a specific portion of the membrane surface, as illustrated in Figure 9b. Only in some regions does increased shear stress result in dead zones in areas with low shear stress. As a result, sludge accumulates and the membrane becomes clogged, reducing the system’s efficiency. In the V-MBR model, the membrane channels that correspond to the Venturi exits have a higher shear stress distribution than the other channels. However, just like in the S-MBR model, there is no substantial change in shear stress at the membrane surface, and the local shear stress in each channel is more uniform.

**Figure 11:**
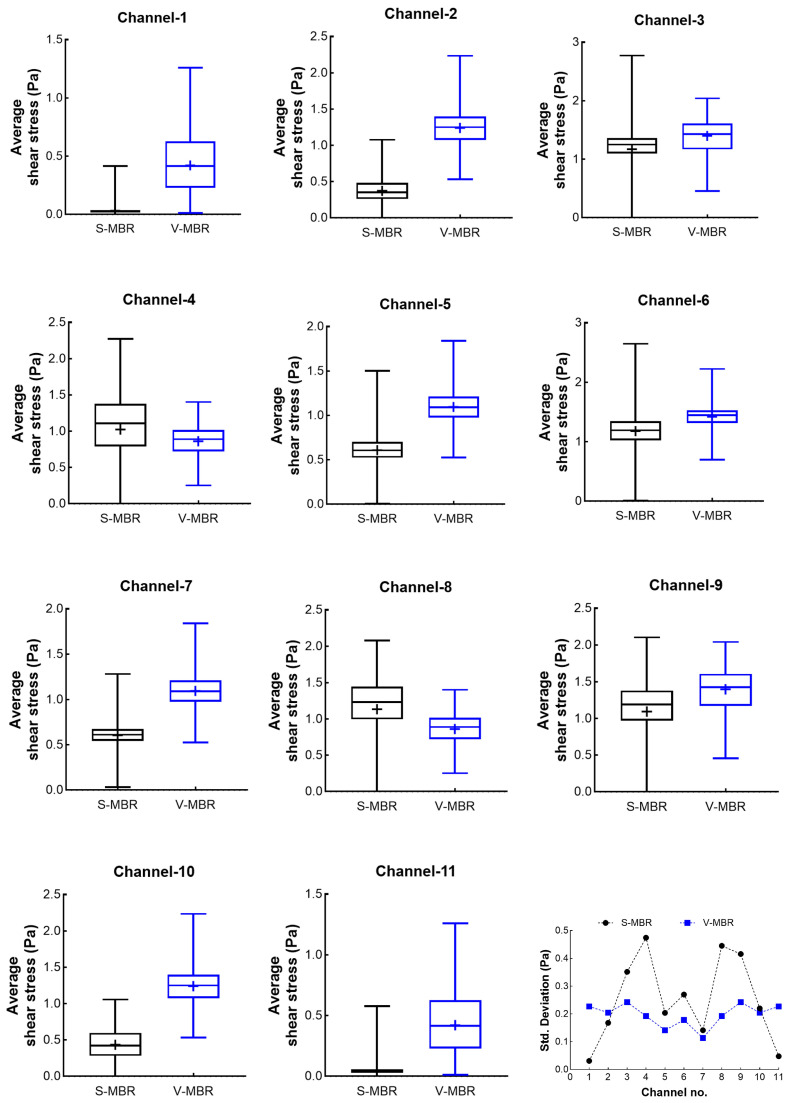
Comparison of shear stress values obtained in the membrane channels for the S-MBR and V-MBR models using box plots.

The shear stress distribution between 1 and 1.5 Pa was visualized and compared using a color scale on the shear stress contours. Because statistical variables such as minimum, maximum, and variance could not be read from the stress contours, statistical analysis was used to appropriately evaluate these values. For this reason, shear stress data from the membrane channels of the two models is to be exported. Approximately 20,000 shear stress values measured at nodes along each membrane surface were transformed to box plots and compared (Figure 11). Figure 9c shows a schematic illustration of the surface where shear stress values were recorded in the membrane channels. Box plots show the median value represented by the central line, the mean value represented by the plus symbol inside the box, the interquartile range (IQR) represented by the box boundaries, and the horizontal lines representing the minimum and maximum values of the shear stress distribution. Furthermore, Table 3 presents comparative statistical values such as minimum, maximum, median, mean, standard deviation (SD), coefficient of variation (CV), and skewness for each membrane channel. When Figure 11 and Table 3 are evaluated together, the following results are obtained.

In the S-MBR model, the shear stress in channel-1 and channel-11 is very low and does not vary significantly. For the S-MBR model, a narrower variance is expected since very low shear stresses occur in these channels. In the V-MBR model, the average shear stress in the same channels is significantly higher and has a wider distribution. The variance of shear stress in channel-2 and channel-10 is very close to each other. However, it is observed that the V-MBR model produces higher shear stresses with similar variance. In channel-4 and channel-8, the average shear stress in the S-MBR model is higher than in the V-MBR model; however, the variance is wider. In other membrane channels, generally the V-MBR model obtained higher average shear stresses with a smaller variance. Except for channel-1 and channel-11, the minimum shear stress did not reach zero in the V-MBR model. In the S-MBR model, zero and near-zero stresses occurred in all channels. This indicates that high stress occurs at one point on the membrane surface while low stress occurs at another point. This difference between the two models reflects the effect of flow dynamics on shear stress.

As a result, the V-MBR model produced higher shear stresses than the S-MBR model in almost all channels and showed close variation in 11 membrane channels. While CV values in S-MBR ranged from 30% to 96%, CV values in V-MBR ranged from 12% to 54%. The skewness value in V-MBR is closer to zero. This indicates that the V-MBR system produced higher shear stress magnitudes, lower variability, and more homogeneous distributions across all channels. These findings suggest that the V-MBR has more homogeneous flow characteristics from a hydrodynamic perspective and, consequently, has a higher potential for reducing fouling compared to the S-MBR. The results obtained in this study could not be validated by experimental studies due to the lack of available laboratory infrastructure. Instead, the validity of the proposed model was compared with the S-MBR model data presented in [19]. Experimental validation studies of the newly developed V-MBR model are planned to be carried out within the scope of future research with the establishment of adequate infrastructure.

In this study, the membrane surface shear stress distributions obtained from the V-MBR and S-MBR systems were compared for 11 different channels, and the Kolmogorov–Smirnov (K-S) and Mann–Whitney nonparametric statistical tests were applied for each channel. The K-S test compares the cumulative distribution functions (CDF) of two independent samples and therefore detects differences not only in the mean difference but also in all characteristics of the distribution, such as shape, spread, location, and symmetry [33]. In the K-S test, the D value represents the largest difference between the two CDFs. The closer this value is to 1, the greater the difference between the distributions. Mann–Whitney (M-W) tests whether the means or ordered distributions of two independent groups are different. It does not require the assumption of normal distribution and is particularly reliable for large data sets [34].

The *p*-value in the K-S and M-W tests was obtained as <0.0001 for all membrane channels. This finding statistically confirms that the shear stress values in the membrane channels are significantly different. The D value obtained for the channels in the K-S test ranges from 0.3754 to 0.9906. This finding indicates that, although there are differences in the degrees of difference between the channels, there is a generally significant high difference. In the M-W test, the difference between the medians obtained according to the Actual and Hodges-Lehmann methods ranged from 0.1772 to 0.8971. When the findings obtained from the K-S and M-W tests are evaluated together, the distributions are significantly different in all channels, both in shape and in central tendency. These results are strong evidence that the hydrodynamic behavior of the two systems is different in all regions (Table 4).

## 4. Conclusions

In this study, a new model (V-MBR) with a micro-Venturi-integrated diffuser structure is developed as an alternative to the S-MBR model proposed by Shen et al. [19]. The details of the analysis results of the S-MBR and V-MBR models are presented with the analysis performed under the same conditions. The focus of the study is to investigate the hydrodynamic effects of the new V-MBR model on the membrane surface.

As a result of the comparative analyses, it was found that the V-MBR model produced approximately 50% higher average shear stress than the S-MBR model. This suggests that, compared to the S-MBR, the V-MBR configuration offers improved membrane surface cleaning, which in turn enhances the overall efficiency of the membrane filtration process because of a higher scouring force.

In particular, the absence of homogeneous shear stress on the membrane surface despite increasing energy has been identified as a significant concern in the literature. In the S-MBR concept, air bubbles coming from the diffuser rose in a certain direction before exiting the system through the upper surface. In the V-MBR configuration, the air injected by the Venturi is evenly distributed throughout the reactor. These properties enabled the V-MBR system to provide a uniform shear stress distribution on each membrane surface and between membrane surfaces. Furthermore, the homogeneous distribution of air across a vast region in the MBR might boost oxygen transfer rates by improving air–water interaction. This is corroborated by research in the literature that shows Venturi injectors have a high oxygen transfer rate.

The V-MBR model revealed that the shear stress readings recorded along the membrane surface (about 20,000 readings per membrane surface) differed from zero. This suggests that the system’s effectiveness can be increased by limiting the formation of dead zones on the membrane surface.

## Figures and Tables

**Table 1:** Summary of mesh properties for optimization of mesh independence testing.

Model	Min. Mesh Size [mm]	Max. Mesh Size [mm]	Mesh Type	Mesh Number	Skewness	Aspect Ratio	Average Velocity [m/s]	Average Shear Stress [Pa]
1	0.5	13	Polyhedral	114,162	0.69	11.59	0.1364	0.6052
2	0.4	9	Polyhedral	340,407	0.58	8.21	0.1406	0.6312
3	0.3	7	Polyhedral	575,909	0.50	6.27	0.1433	0.6673
4	0.3	5	Polyhedral	894,025	0.50	6.22	0.1467	0.6787
5	0.2	5	Polyhedral	985,088	0.52	6.83	0.1552	0.6883
6	0.2	4	Polyhedral	1,450,079	0.48	6.09	0.1584	0.6911
7	0.2	3.5	Polyhedral	1,970,998	0.45	5.70	0.1597	0.6920

**Table 2:** Specification of simulation conditions for numerical models.

Simulation Methods and Conditions		Standard Scenario
Models	Phase	Two-phase: liquid–gas
	Multiphase model	Eulerian model
	Turbulent model	Standard k–ε
	Near-wall function	Standard wall functions
Boundary conditions	Inlet-1 (water inlet)	Velocity-inlet (Only V-MBR)
	Inlet-2 (air inlet)	Pressure-inlet
	Outlet-1 (MBR surface)	Degassing
	Outlet-2 (water outlet)	Pressure-outlet (Only V-MBR)
	Membrane surface	Wall
Solution methods	Pressure–velocity coupling	Phase-coupled SIMPLE
	Spatial Discretization for gradient	Least squares cell-based
	Spatial Discretization for momentum	Quick
	Spatial Discretization for volume fraction	Quick
Solution controls	Pressure	0.5
	Intensity	1
	Momentum	0.2
	Turbulent Kinetic Energy	0.8

**Table 3:** Statistical comparison of shear stress distributions in the membrane channels of V-MBR and S-MBR systems.

		Shear Stress (Pa)								
Channel No	Model	Number of Values	Minimum	Maximum	Median	Mean	Std. Deviation	Coefficient of Variation	Skewness	Kurtosis
1	S-MBR	20,809	0.00013	0.41480	0.02553	0.03238	0.03112	96.09%	3.85000	24.54000
	V-MBR	21,255	0.01123	1.26000	0.41580	0.42070	0.22700	53.95%	0.01658	−1.16200
2	S-MBR	20,856	0.00064	1.07600	0.35130	0.37420	0.16810	44.91%	0.27760	−0.10200
	V-MBR	21,148	0.53090	2.23500	1.24800	1.24000	0.20480	16.52%	0.01591	−0.69750
3	S-MBR	20,893	0.00326	2.77300	1.25100	1.17200	0.35140	29.99%	−1.25500	2.84900
	V-MBR	21,109	0.45510	2.04100	1.42800	1.39800	0.24240	17.34%	−0.17010	−1.14900
4	S-MBR	20,991	0.00101	2.27500	1.10800	1.02500	0.47450	46.30%	−0.61560	−0.47240
	V-MBR	20,934	0.25310	1.40300	0.88940	0.86140	0.19240	22.34%	−0.32430	−0.88420
5	S-MBR	21,184	0.00446	1.50200	0.60630	0.60790	0.20370	33.51%	−0.18780	1.43000
	V-MBR	21,172	0.52630	1.84100	1.09100	1.09600	0.14140	12.90%	0.05714	−0.74080
6	S-MBR	21,216	0.00720	2.65000	1.19100	1.17800	0.27000	22.92%	−0.37200	3.22600
	V-MBR	20,926	0.69720	2.22200	1.44400	1.42100	0.17840	12.55%	−0.16770	0.62580
7	S-MBR	21,184	0.00446	1.50200	0.60630	0.60790	0.20370	33.51%	−0.18780	1.43000
	V-MBR	21,172	0.52630	1.84100	1.09100	1.09600	0.14140	12.90%	0.05714	−0.74080
8	S-MBR	20,991	0.00101	2.27500	1.10800	1.02500	0.47450	46.30%	−0.61560	−0.47240
	V-MBR	20,934	0.25310	1.40300	0.88940	0.86140	0.19240	22.34%	−0.32430	−0.88420
9	S-MBR	20,893	0.00326	2.77300	1.25100	1.17200	0.35140	29.99%	−1.25500	2.84900
	V-MBR	21,109	0.45510	2.04100	1.42800	1.39800	0.24240	17.34%	−0.17010	−1.14900
10	S-MBR	20,856	0.00064	1.07600	0.35130	0.37420	0.16810	44.91%	0.27760	−0.10200
	V-MBR	21,148	0.53090	2.23500	1.24800	1.24000	0.20480	16.52%	0.01591	−0.69750
11	S-MBR	20,809	0.00013	0.41480	0.02553	0.03238	0.03112	96.09%	3.85000	24.54000
	V-MBR	21,255	0.01123	1.26000	0.41580	0.42070	0.22700	53.95%	0.01658	−1.16200

**Table 4:** Comparison of statistical test results for each membrane channel in the S-MBR and V-MBR models.

	Kolmogorov–Smirnov Test		Mann–Whitney Test		
Channel No	*p* Value	D	*p* Value	Difference Between Medians	
				Actual	Hodges–Lehmann
1	<0.0001	0.8787	<0.0001	0.3903	0.3832
2	<0.0001	0.9906	<0.0001	0.8971	0.8690
3	<0.0001	0.3754	<0.0001	0.1772	0.2005
4	<0.0001	0.4190	<0.0001	0.2183	0.2302
5	<0.0001	0.8804	<0.0001	0.4848	0.4805
6	<0.0001	0.4576	<0.0001	0.2533	0.2358
7	<0.0001	0.8804	<0.0001	0.4848	0.4805
8	<0.0001	0.4190	<0.0001	0.2183	0.2302
9	<0.0001	0.3754	<0.0001	0.1772	0.2005
10	<0.0001	0.9906	<0.0001	0.8971	0.8690
11	<0.0001	0.8787	<0.0001	0.3903	0.3832

## Data Availability

The original contributions presented in this study are included in the article. Further inquiries can be directed at the corresponding author.
